# Linagliptin unmasks specific antioxidant pathways protective against albuminuria and kidney hypertrophy in a mouse model of diabetes

**DOI:** 10.1371/journal.pone.0200249

**Published:** 2018-07-06

**Authors:** Netanya Y. Spencer, Zhihong Yang, Jensyn Cone Sullivan, Thomas Klein, Robert C. Stanton

**Affiliations:** 1 Joslin Diabetes Center, Boston MA, United States of America; 2 Harvard Medical School, Boston MA, United States of America; 3 Beth Israel Deaconess Medical Center, Boston MA, United States of America; 4 Boehringer-Ingelheim, Ingelheim am Rhein, Germany; Baker IDI Heart and Diabetes Institute, AUSTRALIA

## Abstract

**Background:**

Dipeptidyl peptidase-4 (DPP-4) inhibitors may have protective effects on diabetic kidney disease (DKD) via specific antioxidant pathways. The DPP-4 inhibitor, linagliptin, was evaluated with the hypothesis that DPP-4 inhibition would ameliorate the development of DKD in a glucose-independent manner by altering specific antioxidant function.

**Methods:**

DBA/2J mice (a well-characterized model of DKD) and glucose 6-phosphate dehydrogenase (G6PD) deficient mice (a model of impaired antioxidant function) were evaluated. Diabetes was induced by streptozotocin. Mice were divided into: diabetic (DM), diabetic+linagliptin (DM+Lina), and non-diabetic control and treated for 12 weeks.

**Results:**

In DBA/2J mice, there was no difference in body weight and blood glucose between DM and DM+Lina groups. Linagliptin ameliorated albuminuria and kidney hypertrophy in DM DBA/2J mice and specifically increased the mRNA and protein levels for the antioxidants catalase and MnSOD. In G6PD deficient mice, however, increases in these mRNA levels did not occur and linagliptin renoprotection was not observed. Linagliptin also ameliorated histological trends toward mesangial expansion in wild-type mice but not in G6PD deficient mice.

**Conclusions:**

Linagliptin renoprotection involved glucose-independent but antioxidant-enzyme-system-dependent increases in transcription (not just increased protein levels) of antioxidant proteins in wild-type mice. These studies demonstrate that an intact antioxidant system, in particular including transcription of catalase and MnSOD, is required for the renoprotective effects of linagliptin.

## Introduction

Diabetic kidney disease (DKD) continues to rise at epidemic rates throughout the world despite current treatments [1, 2]. There is a clear need to determine whether existing drugs may have renal protective actions and to discover new targets for drug development. DPP-4 inhibitors (such as linagliptin, saxagliptin, sitagliptin, etc.) have shown promise for providing renal protection in animal models [3–5] and humans [6–8]. Whether DPP-4 inhibitors are ultimately useful in people will be determined by ongoing large studies [9]. Regardless of the success of the large clinical trials, understanding the mechanisms by which DPP-4 inhibitors provide renal protection in animals may provide new targets for drug development.

With regards to renoprotective mechanisms, animal studies with DPP4 inhibitors have shown improved pathologic markers such as prevention of glomerulosclerosis and interstitial fibrosis [3, 4, 10]. The mechanisms underlying renoprotection appear to be both GLP-1 dependent and independent [11] as well as independent of the glucose-lowering effect [12, 13]. Renoprotection in animals is mediated at least in part by decreasing oxidative stress [14, 15]. Considering the central role reactive oxygen species (ROS) play in development and progression of DKD, it is important to know how linagliptin improves oxidative stress.

Oxidative stress occurs due to a combination of increased production of ROS, decreased activities of antioxidants, and dysregulated subcellular localization of ROS [16]. In this study, a role for linagliptin in regulating antioxidant enzymes was explored. The hypothesis was that linagliptin would improve oxidative stress in a mouse model of DKD by enhancing antioxidant enzyme actions. The results showed that linagliptin renal protection from diabetes increased the essential antioxidants catalase and MnSOD.

## Materials and methods

### Reagents

Rabbit polyclonal antibody to catalase was purchased from Abcam (Cambridge, MA. # Ab16731). Mouse monoclonal antibody to beta actin was purchased from Proteintech (Rosemont, IL. # 66009-1-Ig). Rabbit polyclonal antibody to MnSOD was purchased from Enzo Life Science (Farmingdale, NY, # ADI-SOD-110-F). Streptozotocin (STZ) was obtained from Sigma-Aldrich (St. Louis, MO). Linagliptin was provided by Boehringer Ingelheim (Ingelheim, Germany) with a material transfer agreement. The enzymatic immunoassay kits for determining urinary albumin and creatinine were purchased from Exocell (Philadelphia, PA).

### Animals

All protocols for animal use and euthanasia were approved by the Animal Care Committee of the Joslin Diabetes Center, Harvard Medical School, and are in accordance with National Institutes of Health (NIH) guidelines. Eight-week-old male DBA/2J mice were purchased from The Jackson Laboratory (Bar Harbor, ME). The G6PD-deficient mouse model has been previously described. Briefly, this strain was recovered in the offspring of 1-ethyl-nitrosourea-treated male mice on a C3H murine background by Pretsch et al[17]. Sequencing showed afterwards that there is a single-point mutation (A to T transversion) in the 5’ splice site consensus sequence at the 3’ end of G6PD exon 1 [18]. For our experiments, the mice were bred at Harvard Medical School from frozen embryos obtained from the Medical Research Council (Harwell, U.K.). Because G6PD is an X-linked gene, heterozygotes and homozygotes are female and hemizygotes are male. Wild-type C3H control mice and hemizygous G6PD-deficient male mice were studied. Animals were genotyped and characterized as described previously[19, 20]. Mice were injected with streptozotocin (STZ) to induce diabetes and DKD. Mice received daily streptozotocin injections intraperitoneally (40 mg/kg made freshly in 0.1 mol/L citrate buffer, pH 4.5) for 5 consecutive days, whereas mice in the control group were injected with equal volume of citrate buffer[21, 22]. Two weeks after STZ injection, mice with blood glucose levels > 250mg/dL were confirmed as valid diabetes models. The diabetic mice were divided into two groups that received either linagliptin or placebo treatment (83ppm in chow diet, PharmaServ Inc., Framingham, MA). Blood glucose was measured using a Bayer glucose meter weekly. Body weight was monitored weekly. 12 weeks after beginning linagliptin treatment, mice were sacrificed. On the day of sacrifice, mice were weighed and anesthetized with isoflurane. Blood was collected into EDTA tubes by cardiac puncture. Both kidneys were perfused with a PBS solution to clear blood from the kidneys, then rapidly removed, decapsulated, and weighed, as described previously[23]. Spot urine samples were collected 2 days before sacrifice.

### Real-time PCR

The mRNA expression of catalase and MnSOD from renal cortex of mice was examined by quantitative Real-time PCR procedures (Applied Biosystems, Grand Island, NY) and normalized to 36B4. PCR primers used in the study are listed in S1 Table.

### Western blotting

Catalase and MnSOD protein expression were measured by Western blot. In brief, lysates of kidney cortex were resolved by 4–20% SDS-PAGE gel and then transferred to nitrocellulose membranes. Membranes were blocked with 5% skim milk solution at room temperature for 1 h and then incubated with 1:2000 dilution of a polyclonal rabbit anti-catalase antibody, and 1:10000 of a polyclonal rabbit anti-MnSOD antibody overnight in a cold room. Membranes were washed and incubated with 1:5000 dilution of a goat anti-rabbit antibody. Immunodetection was performed with an ECL system (Cell Signaling).

### DPP4 activity

DPP4 activity was analyzed as previously described [24].

### Histology

Kidney specimens were fixed in formalin and embedded in paraffin. Sections of each specimen were stained per routine laboratory custom with hematoxylin/eosin (H&E) and periodic acid Schiff stain (PAS). Mesangial expansion was quantified by the method outlined in Waasdorp *et al* [25]. Briefly, 50 glomeruli per mouse were scored as either normal or deviated. Mesangial expansion, appearing as clusters of more than three mesangial cells, defined the glomeruli that were scored as deviated. In other words, when three or more deep purple mesangial cell nuclei were found clustered together in a plane, without clear demarcation of plasma membranes between nuclei, these glomeruli were scored as deviated. Furthermore, deviated glomeruli also demonstrated large anuclear pink areas in PAS-stained specimens with the ratio of anuclear area size to nuclear diameter being markedly greater than in nondiabetic glomeruli.

### Statistical analysis

Data were expressed as mean ± SD. Student t tests were performed for comparison of two groups. GraphPad Prism software (Ver 7.0f) (La Jolla, CA) was used for the statistical analysis. A value of P <0.05 was considered significant.

## Results

S1 Fig shows the protocol for all studies. Mouse models included the well-characterized DBA/2J wild-type (WT) model of DKD [26] as well as C3H WT and G6PD deficient (G6PD^-^) mice. G6PD^-^ mice are on C3H, not DBA/2J background. G6PD^-^ mice have about 15% of WT G6PD function. Streptozotocin (STZ) was injected for 5 days to induce diabetes. Diabetic mice were treated with vehicle or linagliptin for 12 weeks. Then mice were sacrificed and kidneys were used for analysis. The study was stopped at 12 weeks as the STZ model does not demonstrate decreased blood sugar in response to DPP4 inhibition; hence prolonged high sugars would lead to early mortality. Non-diabetic (NDM) DBA/2J mice had normal body weights and blood sugar levels (S2 Fig). Diabetic mice with or without linagliptin had similar body weights and blood sugars. Therefore, any effect of linagliptin is independent of blood sugar level. Similar results were seen in G6PD^-^ mice and wild-type C3H controls (S3 Fig). DPP-4 activity was significantly and similarly inhibited in all mouse models studied (S4 Fig).

**Fig 1:**
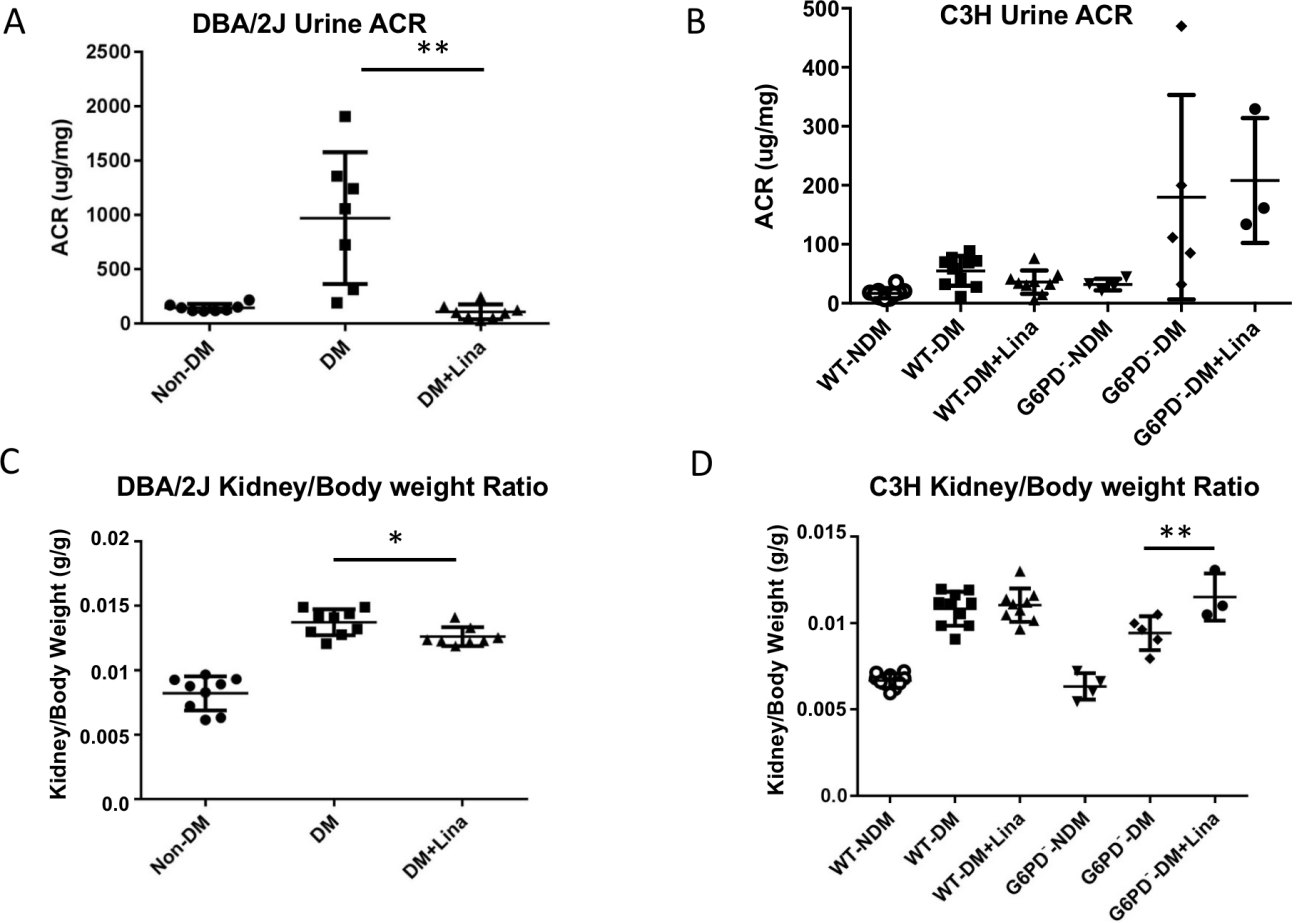
Linagliptin ameliorates albuminuria and kidney hypertrophy in DBA/2J diabetic mice but not in G6PD^-^ mice at 12 weeks. (A, B): Urine albumin/Creatinine Ratio (ACR) in DBA/2J (A) or in C3H WT and G6PD^-^ (B), with or without linagliptin treatment as indicated. Spot urine was collected 2 days before sacrifice. Urine albumin and creatinine were measured. Urine ACR was significantly decreased in the DBA/2J linagliptin group (A). There was a trend of decreasing of urine ACR in WT-DM+Lina group (p = 0.088), but there was no change in G6PD deficient diabetic mice treated with linagliptin (B). (C,D): Kidney/body weight ratio in DBA/2J (C) or in C3H WT and G6PD^-^ (D), with or without linagliptin treatment as indicated. Kidney/body weight ratio was decreased significantly in DBA/2J (C) but increased significantly in G6PD^-^ (D). * *p*<0.05, ** *p*<0.0001 (n = 3–10, mean ± SD, t test).

Albuminuria is observed in DKD. Diabetic DBA/2J mice exhibited increased urine albumin-creatinine ratio (ACR) at the end of 12 weeks compared to nondiabetic controls. Linagliptin treatment lowered urine ACR in diabetic DBA/2J mice (Fig 1A). Linagliptin trended to decrease urine albumin levels in WT C3H diabetic mice (p = 0.08) but not in diabetic G6PD^-^ mice (Fig 1B).

A common finding in DKD is kidney hypertrophy. Diabetic DBA/2J mice develop kidney hypertrophy as determined by kidney weight–to–body weight ratio (Fig 1C) [26]. Diabetic DBA/2J mice treated with linagliptin had partial amelioration of renal hypertrophy (Fig 1C). However, linagliptin significantly worsened hypertrophy in G6PD^-^ mice (Fig 1D), suggesting an uncompensated effect of linagliptin in the absence of the antioxidant pathways. Strain differences amongst mice are very common; linagliptin did not ameliorate kidney hypertrophy in diabetic WT C3H mice.

**Fig 2:**
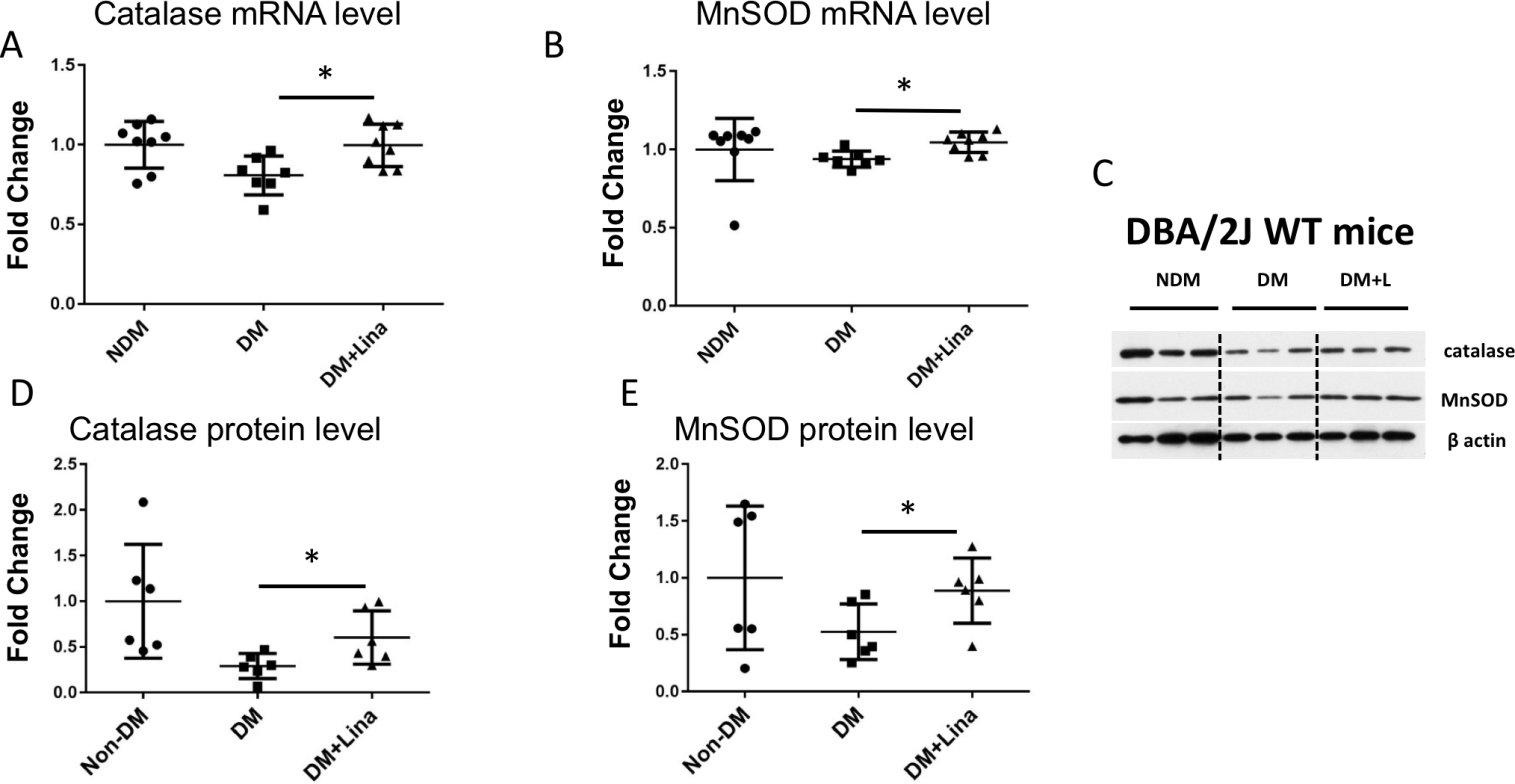
Linagliptin increases catalase and MnSOD mRNA and protein level in DBA/2J mouse kidney. Catalase and MnSOD mRNA and protein expression were analyzed in renal cortex tissue. Data were compared between diabetic mice fed with/without linagliptin. A, B) Catalase and MnSOD mRNA level increased significantly in DBA2/J mice treated with linagliptin. (C) Representative western blot of renal cortex tissue. (D,E) Quantification of western blots using ImageJ. Catalase and MnSOD protein expression increased with linagliptin treatment. * *p*<0.05 (n = 7–8 for qPCR, n = 6 for western blot. mean ± SD, t test).

**Fig 3:**
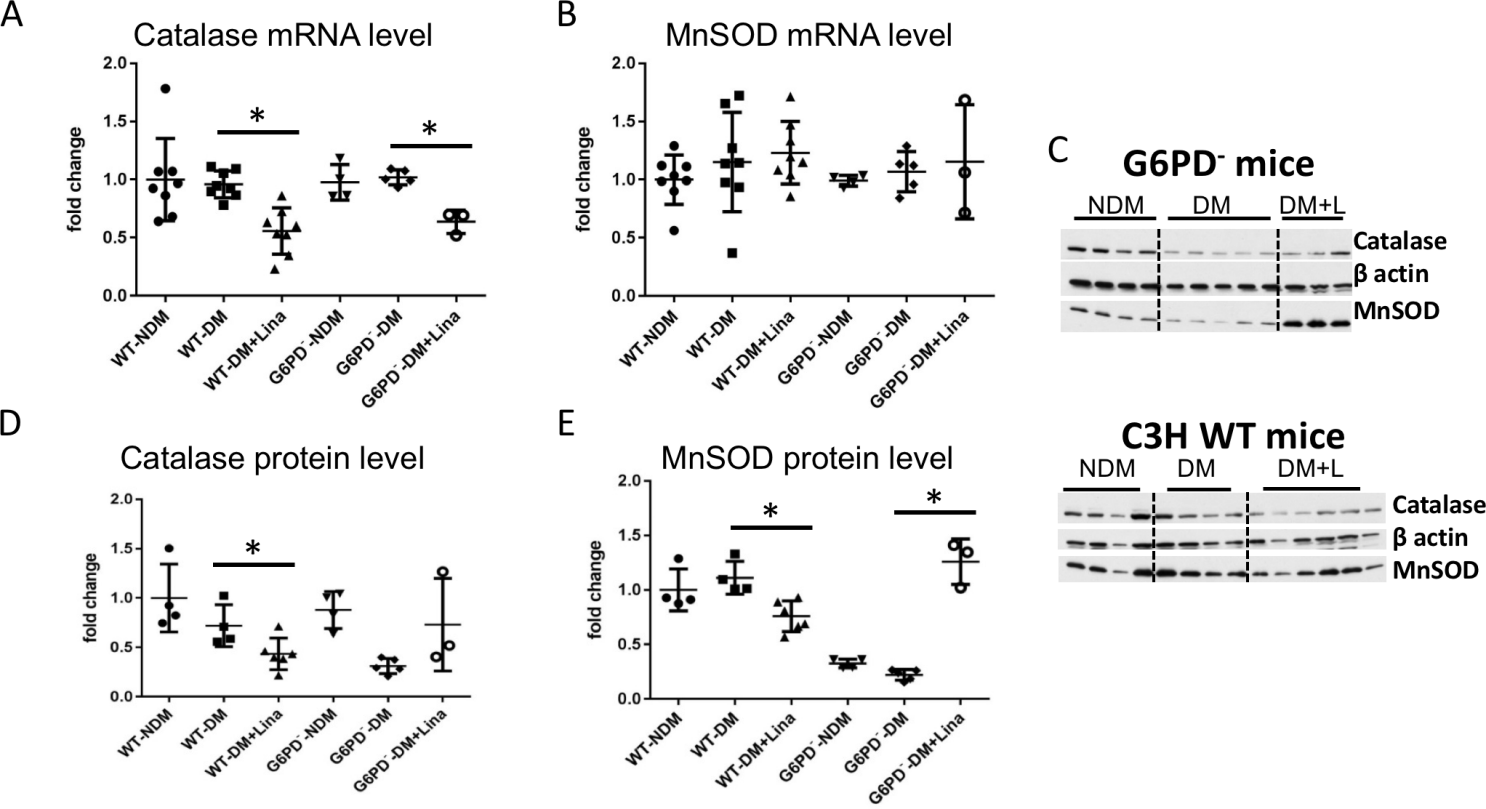
Linagliptin does not increase catalase and MnSOD mRNA but trends to increase catalase protein level while significantly enhancing MnSOD protein level in G6PD^-^ mouse kidney. Catalase and MnSOD mRNA and protein expression were analyzed in renal cortex tissue. Data were compared between diabetic mice fed with/without linagliptin. (A,B) Catalase and MnSOD mRNA decreased (catalase) or did not change (MnSOD) in mice treated with linagliptin. (C) Representative western blots of renal cortex tissue. (D,E) Quantification of western blots using ImageJ. Catalase protein expression decreased with linagliptin treatment in WT C3H and only trended to increase in G6PD^-^. MnSOD protein expression decreased with linagliptin treatment in WT C3H but significantly increased in G6PD^-^. * *p*<0.05 (n = 7–8 for qPCR, n = 6 for western blot. mean ± SD, t test).

The cellular antioxidant system is composed primarily of catalase, the glutathione system, G6PD, and superoxide dismutases. Protein and mRNA levels of enzymes from these pathways were evaluated from the cortex of the sacrificed mice. Linagliptin upregulated both mRNA and protein of two enzymes, catalase and MnSOD, in DBA/2J kidney cortex (Fig 2). In G6PD^-^ mice on linagliptin, catalase protein only trended upward, but MnSOD protein was very enhanced, whereas antioxidant enzyme mRNA levels did not increase (Fig 3). Thus linagliptin enhances antioxidant protein levels independently of its (possibly G6PD-dependent) enhancement of antioxidant enzyme mRNA transcription. Oddly, linagliptin decreased catalase and MnSOD protein levels in wild-type C3H diabetic mice (Fig 3D and 3E). The genetic background of these mice is not fully characterized, and various factors may have influenced this observation. The reasons for these decreases in MnSOD and catalase are not clear and are outside the scope of this study.

Histological changes of DKD in rodent STZ models are often delayed well past the initiation of kidney disease demonstrated by albuminuria and are often not very pronounced [27, 28]. Nevertheless, we were able to observe trends toward increased mesangial expansion consistent with early signs of DKD. Trends toward mesangial expansion appeared in most of the STZ-treated mice, and linagliptin ameliorated these trends only in mice with normal levels of G6PD (S5 and S6 Figs), but not in mice with G6PD deficiency.

## Discussion

Of the molecular mechanisms underlying the development of DKD, increased ROS is likely of central importance. To date, clinical trials on DKD with antioxidants have not been successful, possibly because previous trials employed general antioxidants that were not specifically targeted. Much attention, including clinical trials, has been directed towards sources of increased production of ROS in DKD including such processes as activated NADPH oxidase [29, 30] and enhanced mitochondrial superoxide production [31, 32]. Less attention has been directed to the importance of impaired antioxidant enzyme function even though diabetes mellitus and DKD are associated with this [23, 33]. The entire antioxidant system requires the reductant NADPH [34, 35]. Hence G6PD is of central importance to the regulation of cellular antioxidant capacity. Indeed, decreased G6PD expression and activity has been shown in diabetic conditions in mice [23]. But whether decreased G6PD is the major defect in diabetes as compared to other antioxidant enzymes has not previously been addressed. It is possible that decreases in any one (or more) of the antioxidant enzymes and pathways may be sufficient to lead to increased ROS and resultant DKD. In this study, linagliptin-treated animals had specific increases in catalase and MnSOD suggesting that these antioxidants may be necessary for the renal protective effects of linagliptin. However, without G6PD, they are not sufficient to prevent the development of DKD (Compare Figs 1B and 3E). This emphasizes the importance of an intact antioxidant system, and probably of G6PD, in protection against DKD.

G6PD is the main source of NADPH, on which the entire antioxidant system relies. We reasoned that if linagliptin renal protective effects are not observed in G6PD^-^ mice, we could infer that linagliptin renal protection requires an intact antioxidant pathway. In diabetic DBA/2J mice, linagliptin led to decreased renal hypertrophy (Fig 1C). Renal hypertrophy is a hallmark of DKD. To our knowledge this is the first demonstration of reduction in renal hypertrophy by a DPP-4 inhibitor. Whether linagliptin affects renal hypertrophy in humans has not been evaluated. The worsening of renal hypertrophy in G6PD^-^ mice on linagliptin indicated that linagliptin requires intact antioxidant pathways for renal protection. The lack of protection also against albuminuria in G6PD^-^ mice (Fig 1B) is in agreement with this.

In this study, no insulin was used. It is unknown whether the presence of insulin (and hence better blood sugar control) would have changed the linagliptin effect. At any rate, the effects seen in this study were independent of blood glucose control (all diabetic animals had similar blood sugar levels whether fed linagliptin or not). Studies of type 2 diabetic models such as high fat diet-fed mice would not be able to make this distinction; hence the streptozocin model was ideal for this study. These findings suggest that linagliptin has effects independent of blood glucose that are beneficial in preventing signs of DKD (renal hypertrophy and albuminuria).

Clinical trials have been unable to test outcomes independent of blood glucose. There have been several interesting clinical trials completed recently, that looked at cardiovascular outcomes of DPP4 inhibitors other than linagliptin. These trials included SAVOR-TIMI (which used saxagliptin) [36], TECOS (which used sitagliptin) [37], and EXAMINE (which used alogliptin) [38]. TECOS and EXAMINE did not specifically look at any renal outcomes. SAVOR-TIMI did, however, observe declines in rates of microalbuminuria progression with DPP4 inhibition. This study was over two years long. Because of the nature of clinical trials, it was not possible to determine whether this outcome was driven by reductions in blood pressure, glucose levels, *etc*, or was independent of such effects.

Interestingly, some clinical trials of linagliptin, including MARLINA, which looked at renal outcomes, have been completed [39]. Unlike SAVOR-TIMI, no renal improvements were observed. However, SAVOR-TIMI lasted over two years, but MARLINA was only a 24-week study. Thus it is possible that improvements in renal outcomes might be seen with longer treatment with linagliptin, perhaps by reducing inflammation and fibrosis. On the other hand, a shorter (12-week) study, GUARD, recently demonstrated an albuminuria-reducing effect of gemigliptin, another DPP4 inhibitor [40].

Two clinical trials of linagliptin are currently in progress or have recently been completed, CAROLINA and CARMELINA. CARMELINA was recently completed but the data have not been published yet. This study included measurement of: “Time to the first occurrence of any of the following by adjudication confirmed components: Composite renal endpoint (renal death, sustained end stage renal disease, sustained decrease of 40% or more in estimated glomerular filtration rate).” (https://clinicaltrials.gov/ct2/show/NCT01897532, accessed 10-31-2017). CAROLINA is due to be completed in 2019 and is described online as studying outcomes including “Creatinine, eGFR (MDRD formula), Urinary Albumin.” (https://clinicaltrials.gov/ct2/show/NCT01243424, accessed 10-31-2017). It will be interesting to learn the findings of both of these trials in regards to renal outcomes.

It is not known how linagliptin causes increases in MnSOD and catalase. In this study, linagliptin effects were independent of blood glucose control. Hence another consequence of DPP-4 inhibition must have led to the observed effects. DPP-4 is a multifunction (protease/peptidase and ligand-binding) protein. Further work is needed to elucidate which molecular mechanism targeted by linagliptin leads to increased MnSOD and catalase. The fact that protein and mRNA levels of antioxidant enzymes increased in DBA/2J mice (Fig 2) but only protein levels increased in G6PD^-^ mice (Fig 3) suggests that linagliptin has both G6PD-independent effects on protein expression (either translation or decreased protein ubiquitination/breakdown) and also G6PD-dependent effects on mRNA expression/transcription. In Fig 3, there is no significant difference between WT controls and WT diabetics in terms of MnSOD and Catalase mRNA and protein levels. In G6PD deficient mice, linagliptin did cause an increase in MnSOD but this is medically irrelevant. This is demonstrated by the fact that their kidney-to-bodyweight ratio actually went up (compare Fig 1). This is likely because the antioxidant system is not intact in these animals. The linagliptin-induced increase in MnSOD in G6PD deficient mice is not ultimately beneficial. Therefore, to obtain real kidney benefit from linagliptin treatment, an intact antioxidant system that includes a normal level of G6PD seems to be necessary (although not sufficient).

For enzymatic stability, catalase is NADPH-dependent, but MnSOD is NADPH-independent. This may explain why catalase protein level only trended towards an increase but MnSOD protein level increased dramatically in G6PD^-^ kidneys of mice on linagliptin.

The G6PD-deficient mouse strain is an excellent model of impaired antioxidant function as the resultant decrease in NADPH production leads to an impaired cellular antioxidant system. Linagliptin led to increased renal hypertrophy and no improvement in urine albumin level in diabetic G6PD^-^ mice. These results suggest that antioxidant system deficiency resulted in abrogation of protective effects of linagliptin on diabetes-induced kidney damage. Further mechanistic analysis of DPP-4 substrates in mice may provide new insights into protective and deleterious DKD factors. It is important to note that these mice are a C3H strain, not DBA/2J, as it is well known that there are highly significant mouse strain differences in susceptibility to the development of DKD and to the effectiveness of treatment. The current study does not address whether DKD would be worsened in G6PD deficient diabetic patients on linagliptin. The current study also does not indicate whether linagliptin will improve DKD in humans. Although linagliptin may or may not be useful in the future to treat DKD [39], this study demonstrates that it is already useful to determine specifics of antioxidant pathways in DKD. Linagliptin can point to specific targets for future drug development.

The current study was unable to complete investigations of urine and tissue markers of oxidative damage such as protein carbonyls, oxidized lipids and nucleic acids, or to measure superoxide by electron paramagnetic resonance, or other reactive oxygen species by other means, but these should be studied in the future.

H&E (not shown) and PAS sections 12 weeks after STZ treatment revealed mesangial expansion (S5 and S6 Figs), although this was somewhat limited. The typical histopathological changes associated with diabetic glomerular or tubular nephropathy can take up to 25 weeks to become evident in STZ mouse models [28]. While this does not exclude the model's usefulness, it does suggest that non-histopathological measures (protein to creatinine ratio, kidney to body weight ratio) may be better suited for quantifying extent of disease in this model. However, even with these potential shortcomings, trends toward mesangial expansion were observed in the histological preparations suggestive that the diabetic mice were definitely developing diabetic kidney disease and that linagliptin treatment ameliorated this in mice with normal G6PD levels. Future studies may benefit by carrying similar studies out to 25 weeks to examine histological changes in greater depth.

In the context of diabetes, DPP4 inhibitors have been described as having many effects including antioxidant effects. Organs that may be affected by a number of mechanisms, such as antioxidant mechanisms, include not only the kidney, but also other cardiovascular targets such as heart [41], aorta [42], and endothelial cells [43]. Neurological targets include retina [44] and brain [45]. Thus in addition to renal effects, it will be important in the future to uncover effects of G6PD on DPP4 inhibitors’ actions in many tissues.

In this study, linagliptin unmasked very specific pathways in DKD. Linagliptin will be useful in future studies to reveal molecular targets for renoprotection. These renal protective effects require an intact antioxidant system.

## Supporting information

S1 Fig Timeline of treatment. Mice received daily STZ injections intraperitoneally (40mg/kg) for 5 consecutive days. 2 weeks after the first injection, blood glucose was measured on two different days to confirm diabetes. Then mice were treated with/without linagliptin (83ppm in chow) for 12 weeks. Blood glucose and body weight were measured weekly.
(TIF)

S2 Fig Weekly body weight and random blood glucose levels of DBA/2J mice. Diabetic mice were treated with/without linagliptin (Lina) in the chow diet (83ppm) for 12 weeks. Body weight (a) and random blood glucose (b) were measured weekly. There was no difference in body weight and blood glucose level in diabetic mice with/without linagliptin. (n = 8–9, mean ± SD).
(TIF)

S3 Fig Weekly body weight and random blood glucose levels of wild-type C3H and G6PD deficient mice. Diabetic wild type and G6PD deficient mice (G6PD^-^) were treated with/without linagliptin (Lina) in the chow for 12 weeks. Body weight (a) and random blood glucose (b) were measured weekly. There was no difference in body weight and blood glucose level in diabetic mice with/without linagliptin. WT–wild type, DM–diabetic, NDM–nondiabetic. (n = 3–10, mean ± SD).
(TIF)

S4 Fig Linagliptin inhibits DPP-4 Activity in all tested strains of mice. DPP-4 activity was measured in plasma. Plasma DPP4 activity decreased significantly with linagliptin treatment in DBA/2J mice (a), and WT and G6PD deficient mice (b). ** *p*<0.01. n = 3–10, mean ± SD, t test.
(TIF)

S5 Fig Histology by H&E and PAS demonstrates trends toward DKD in STZ-treated mice and amelioration of DKD only in mice with normal G6PD levels. (a) Quantitation of deviated glomeruli; 50 random glomeruli were analyzed per mouse and the number of deviated glomeruli out of the 50 total analyzed is shown on the graph (see methods section). *p<0.05. n = 3–10, mean ± SEM, t test.
(TIF)

S6 Fig Representative PAS histological images. Left: Normal glomerulus. Right: Deviated glomerulus. Arrow indicates clump of 3 or more nuclei; asterisk indicates an enlarged anuclear area.
(TIF)

S1 Table Primer sequences. These are the PCR primers used in this study.
(TIF)
