# Supplementary figures and images for: Linagliptin unmasks specific antioxidant pathways protective against albuminuria and kidney hypertrophy in a mouse model of diabetes
Source: PLoS One. 2018 Jul 6;13(7):e0200249. doi: 10.1371/journal.pone.0200249 (PMC6034861; doi:10.1371/journal.pone.0200249)

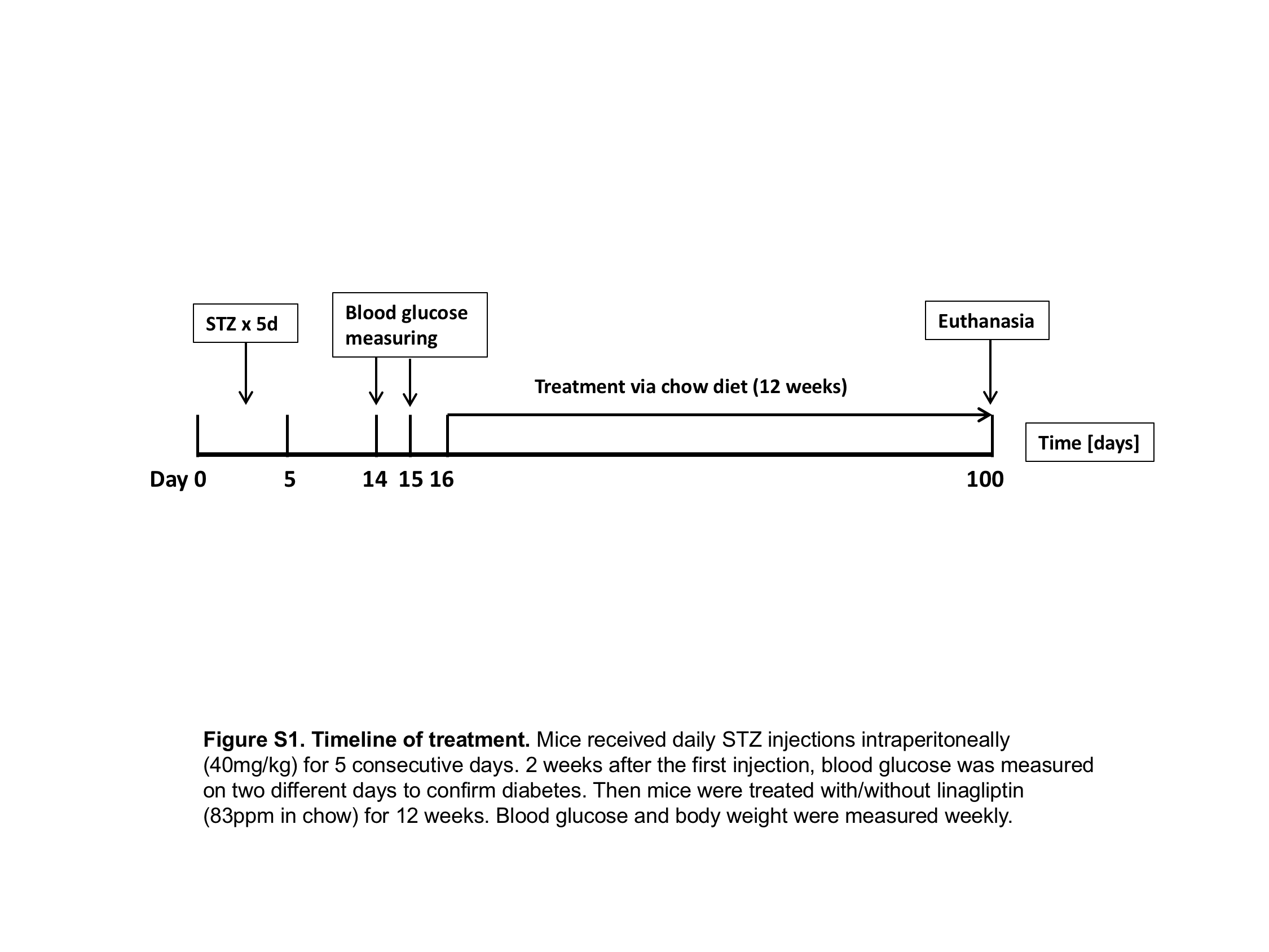

Supplement: S1 Fig — Mice received daily STZ injections intraperitoneally (40mg/kg) for 5 consecutive days. 2 weeks after the first injection, blood glucose was measured on two different days to confirm diabetes. Then mice were treated with/without linagliptin (83ppm in chow) for 12 weeks. Blood glucose and body weight were measured weekly. (TIF) [file pone.0200249.s001.tif]

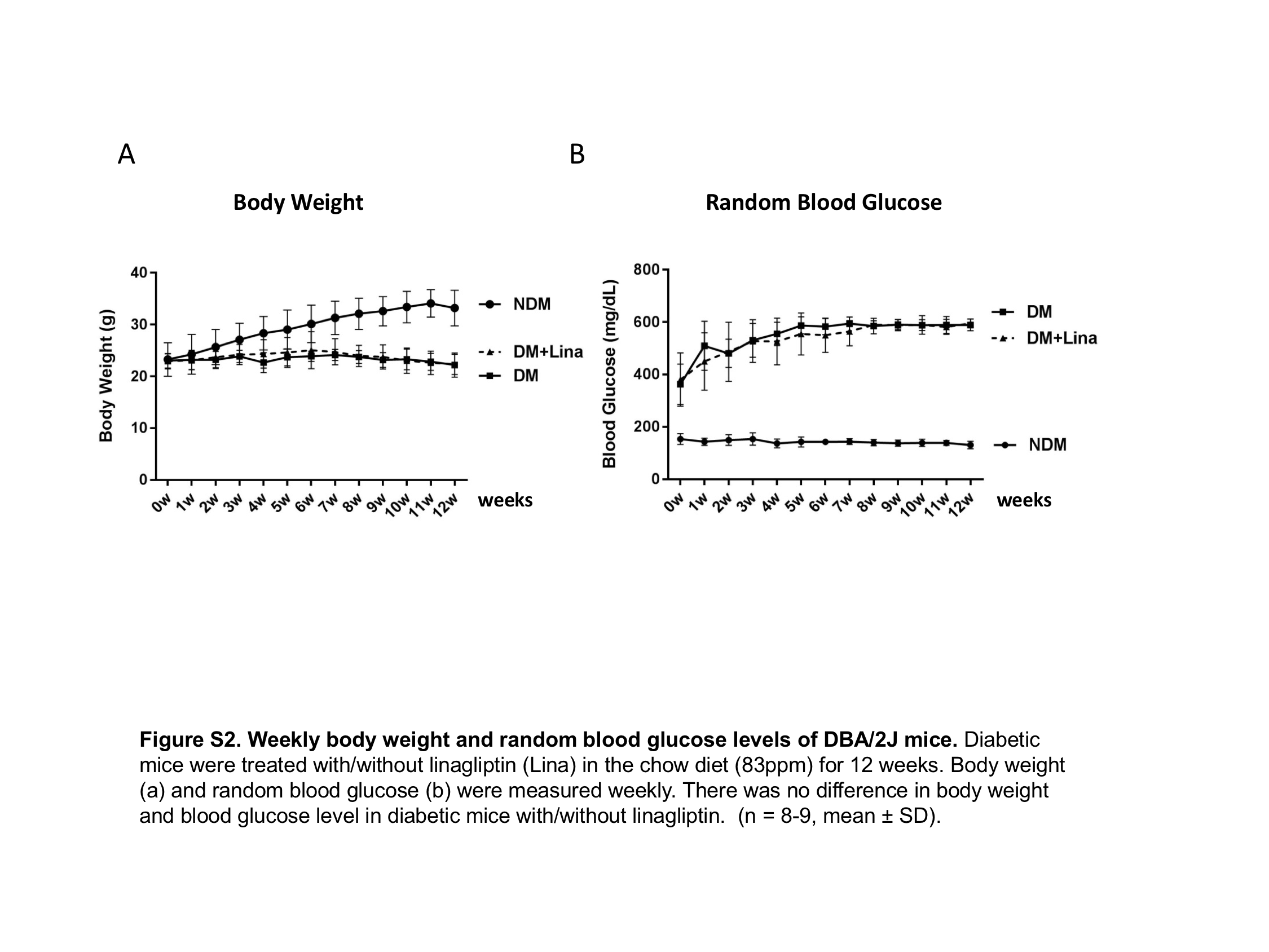

Supplement: S2 Fig — Diabetic mice were treated with/without linagliptin (Lina) in the chow diet (83ppm) for 12 weeks. Body weight (a) and random blood glucose (b) were measured weekly. There was no difference in body weight and blood glucose level in diabetic mice with/without linagliptin. (n = 8–9, mean ± SD). (TIF) [file pone.0200249.s002.tif]

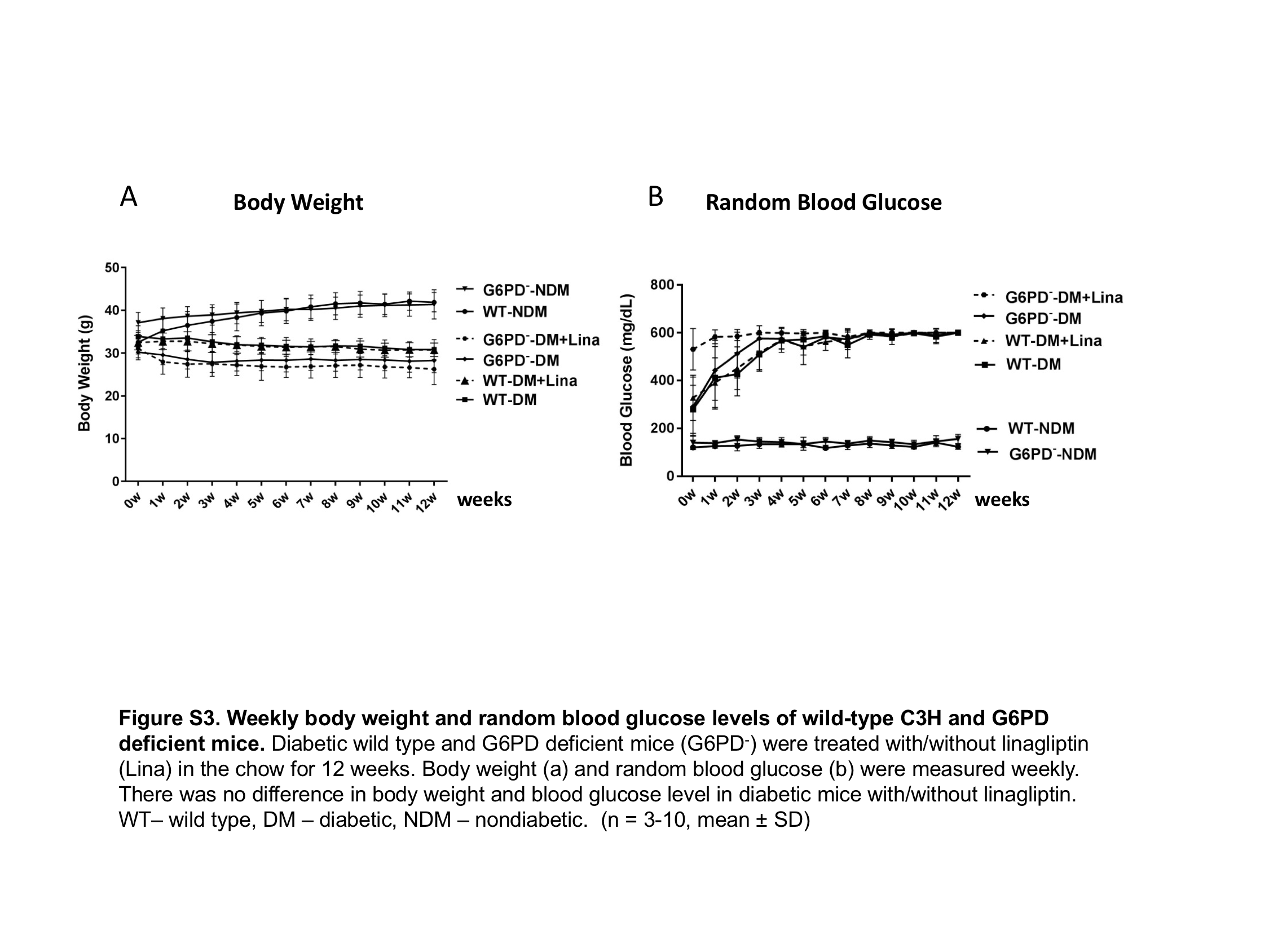

Supplement: S3 Fig — Diabetic wild type and G6PD deficient mice (G6PD-) were treated with/without linagliptin (Lina) in the chow for 12 weeks. Body weight (a) and random blood glucose (b) were measured weekly. There was no difference in body weight and blood glucose level in diabetic mice with/without linagliptin. WT–wild type, DM–diabetic, NDM–nondiabetic. (n = 3–10, mean ± SD). (TIF) [file pone.0200249.s003.tif]

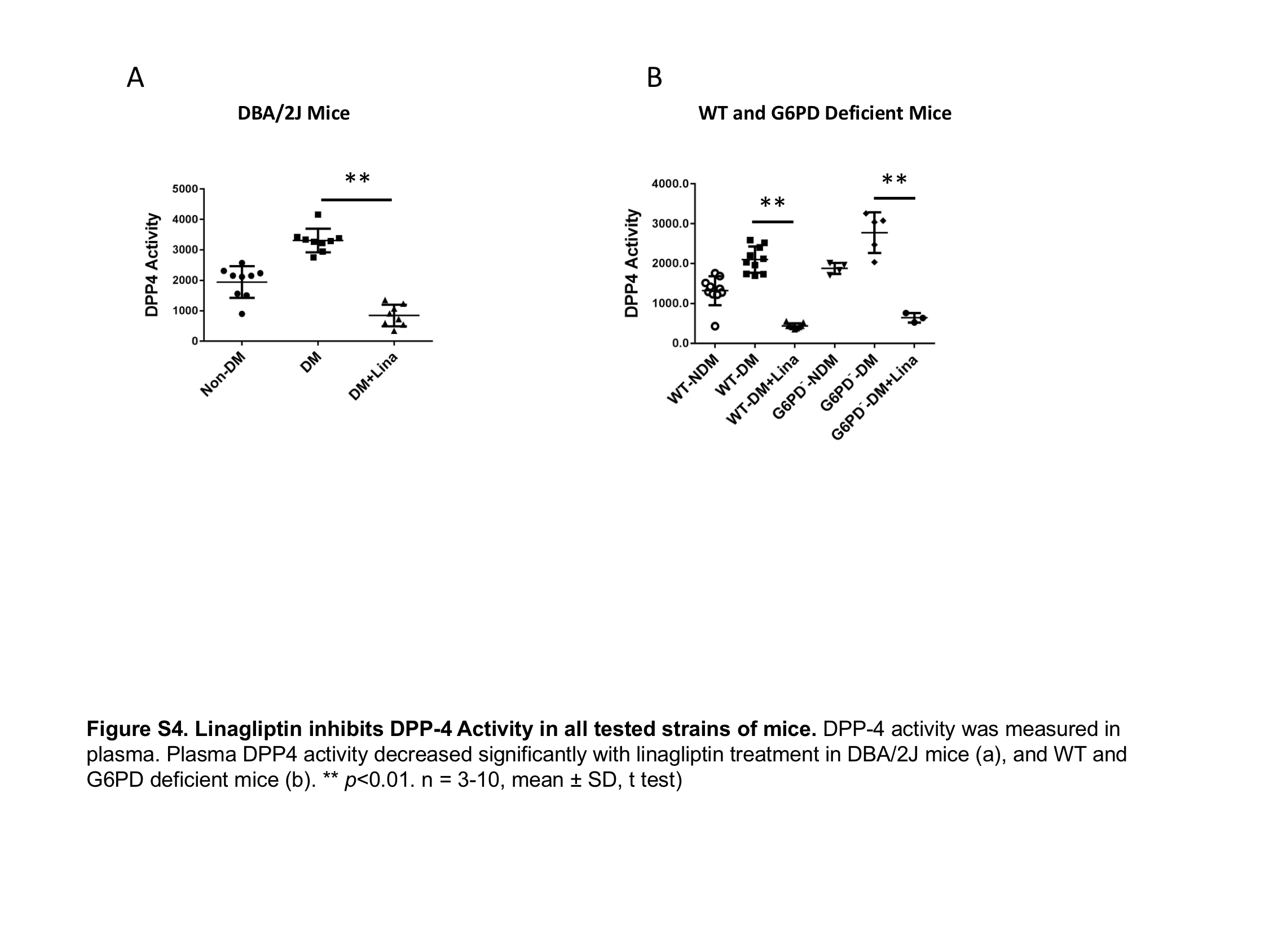

Supplement: S4 Fig — DPP-4 activity was measured in plasma. Plasma DPP4 activity decreased significantly with linagliptin treatment in DBA/2J mice (a), and WT and G6PD deficient mice (b). ** p<0.01. n = 3–10, mean ± SD, t test. (TIF) [file pone.0200249.s004.tif]

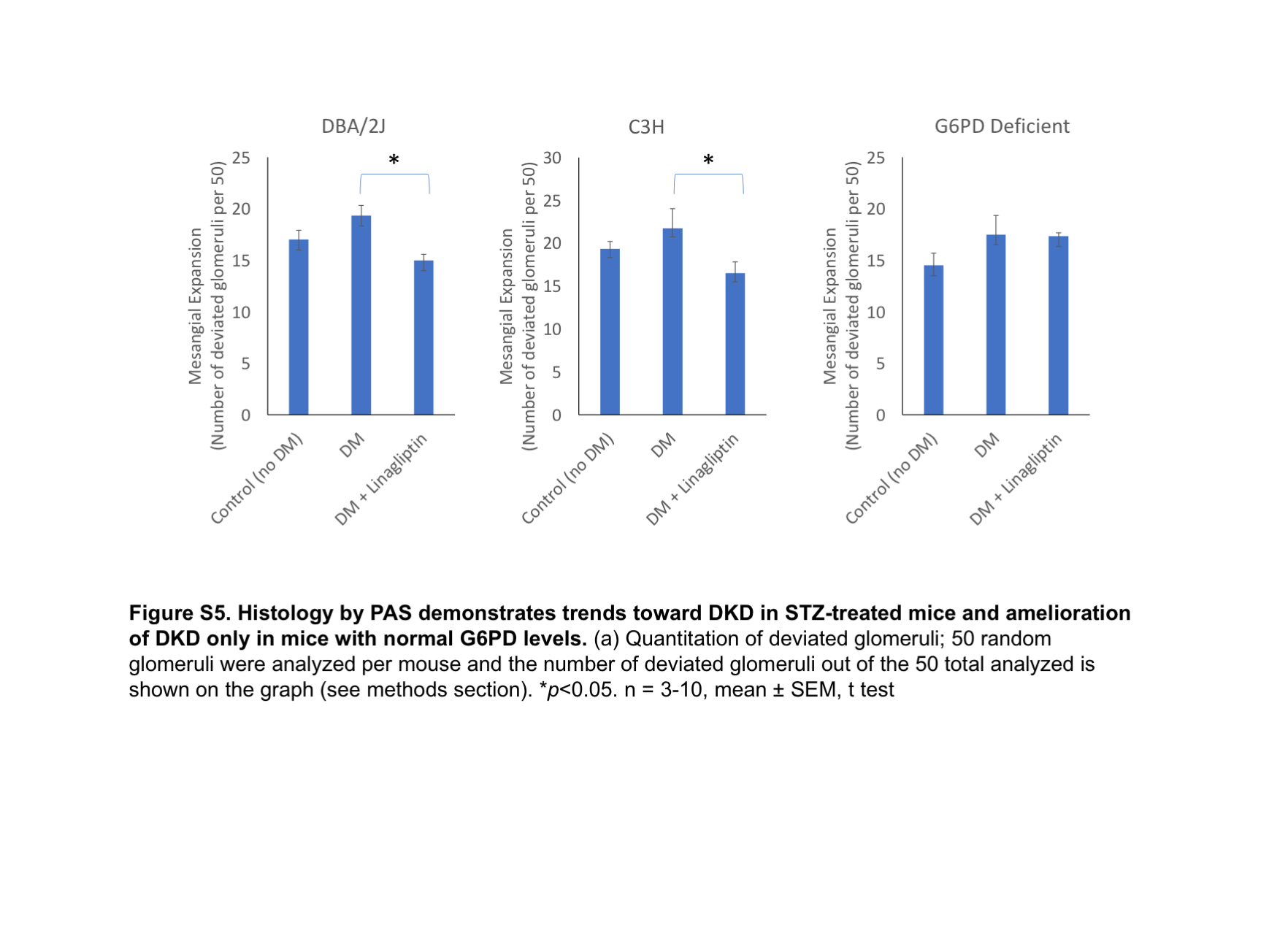

Supplement: S5 Fig — (a) Quantitation of deviated glomeruli; 50 random glomeruli were analyzed per mouse and the number of deviated glomeruli out of the 50 total analyzed is shown on the graph (see methods section). *p<0.05. n = 3–10, mean ± SEM, t test. (TIF) [file pone.0200249.s005.tif]

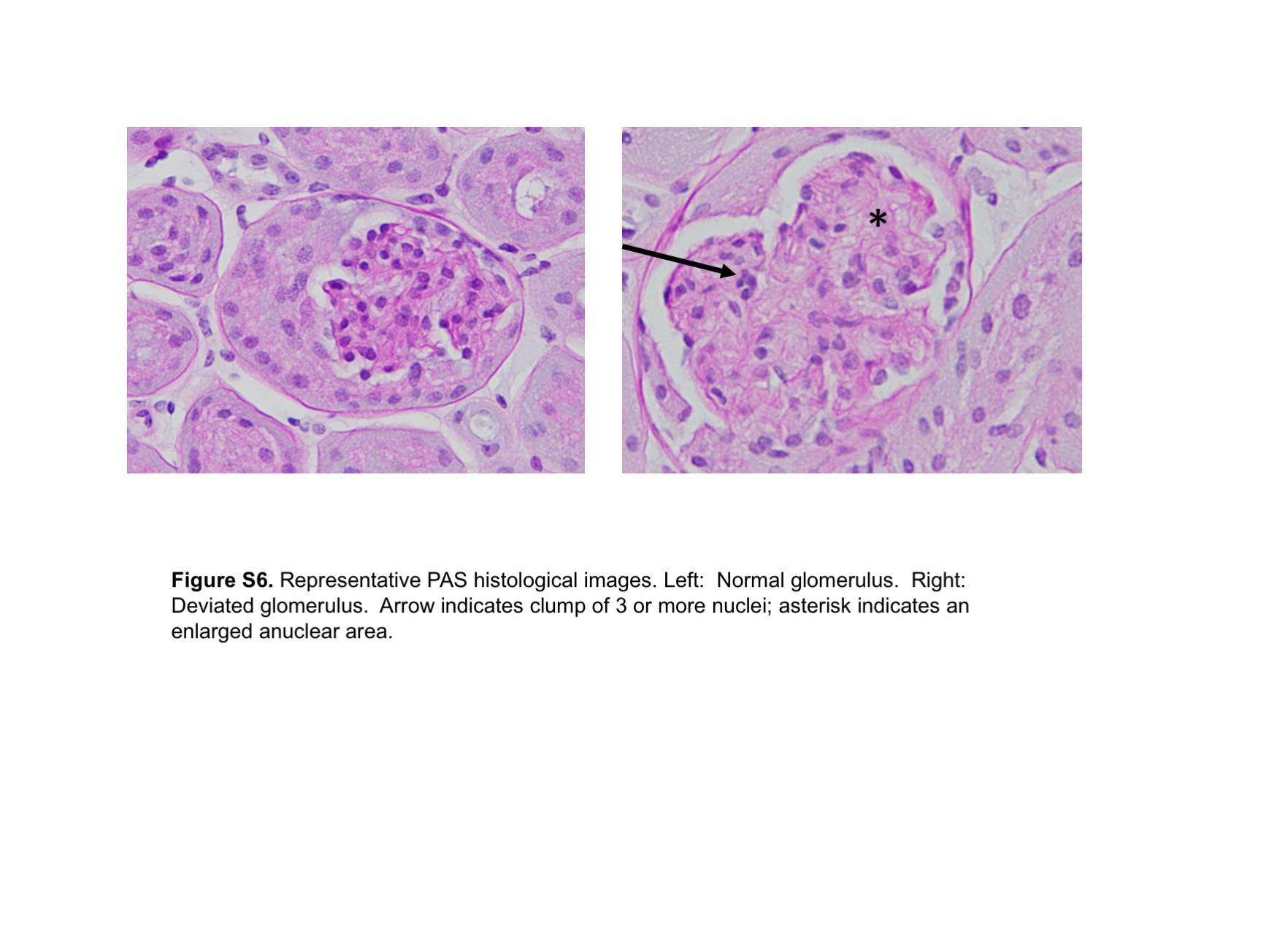

Supplement: S6 Fig — Left: Normal glomerulus. Right: Deviated glomerulus. Arrow indicates clump of 3 or more nuclei; asterisk indicates an enlarged anuclear area. (TIF) [file pone.0200249.s006.tif]

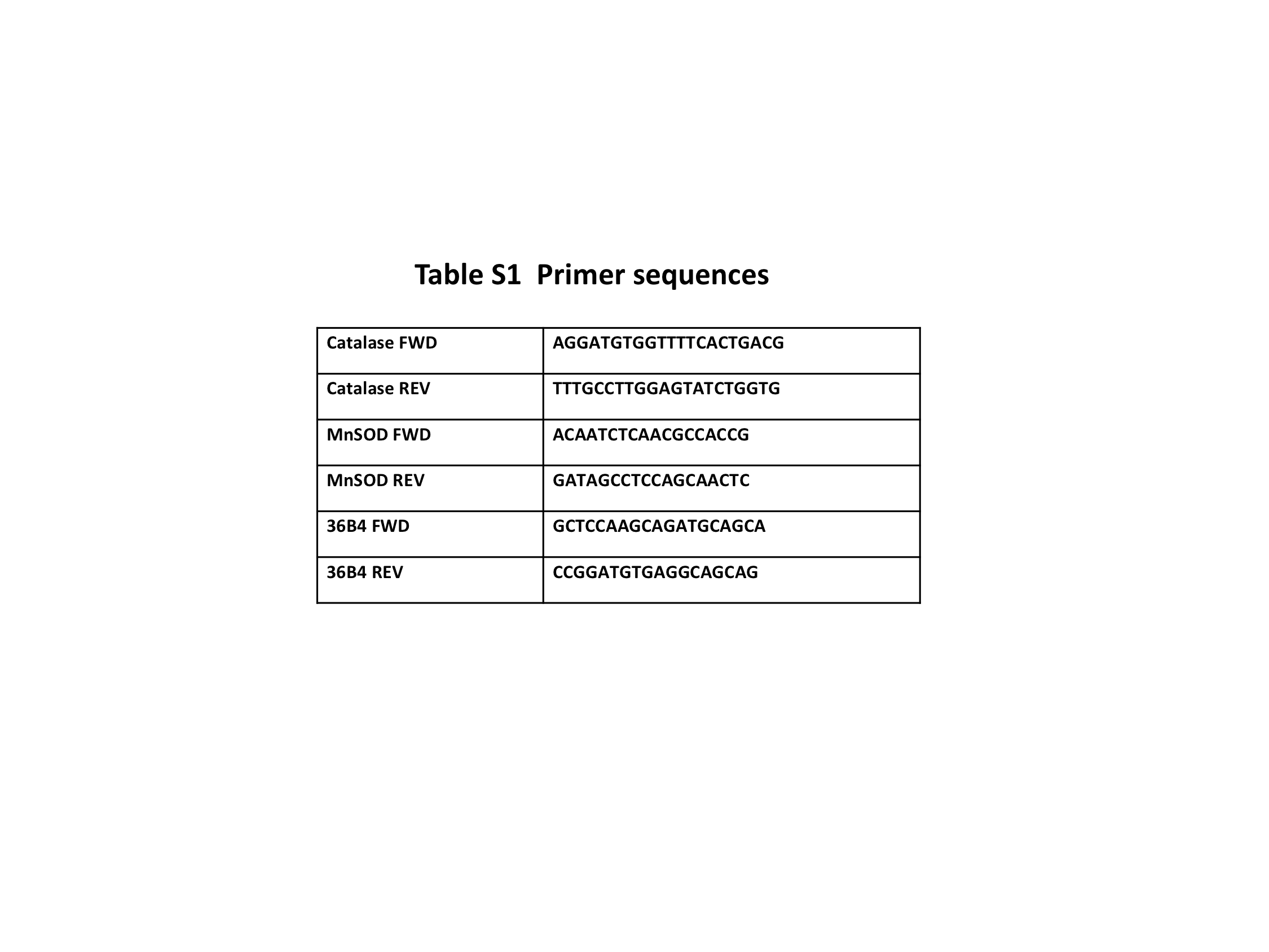

Supplement: S1 Table — These are the PCR primers used in this study. (TIF) [file pone.0200249.s007.tif]
